# Why Percussive Massage Therapy Does Not Improve Recovery after a Water Rescue? A Preliminary Study with Lifeguards

**DOI:** 10.3390/healthcare10040693

**Published:** 2022-04-07

**Authors:** Alejandra Alonso-Calvete, Miguel Lorenzo-Martínez, Alexandra Pérez-Ferreirós, Antonio Couso-Bruno, Eloy Carracedo-Rodríguez, Martín Barcala-Furelos, Roberto Barcala-Furelos, Alexis Padrón-Cabo

**Affiliations:** 1 REMOSS Research Group, Facultade de Ciencias da Educación e do Deporte, Universidade de Vigo, 36005 Pontevedra, Spain; alejalonso@uvigo.es (A.A.-C.); miguel.lorenzo.martinez@uvigo.es (M.L.-M.); tony.cousobruno@gmail.com (A.C.-B.); carracedo54@gmail.com (E.C.-R.); roberto.barcala@uvigo.es (R.B.-F.); 2 Facultade de Fisioterapia, Universidade de Vigo, 36005 Pontevedra, Spain; 3 Facultad de Ciencias de la Salud, Facultad de Ciencias Sociales y Humanidades, Universidad Europea del Atlántico, 39011 Santander, Spain; martin.barcala@uneatlantico.es; 4 Department of Physical Education and Sport Science, Faculty of Sports Sciences and Physical Education, University of A Coruña, 15179 A Coruña, Spain; a.cabo@udc.es

**Keywords:** drowning, lifesaving, recovery modalities, extra-hospital care

## Abstract

The aim of this study was to analyze the effects of percussive massage therapy (PMT) on lifeguards’ recovery after a water rescue, in comparison with passive recovery. **Methods**: A quasi-experimental crossover design was conducted to compare passive recovery (PR) and a PMT protocol. A total of 14 volunteer lifeguards performed a simulated 100 m water rescue and perceived fatigue and blood lactate were measured as recovery variables after the rescue and after the 8-min recovery process. **Results**: There were no differences between PMT and PR in lactate clearance (*p* > 0.05), finding in both modalities a small but not significant decrease in blood lactate. In perceived fatigue, both methods decreased this variable significantly (*p* < 0.001), with no significant differences between them (*p* > 0.05). **Conclusions**: PMT does not enhance recovery after a water rescue, in comparison with staying passive. Despite PMT appearing to be adequate for recovery in other efforts, it is not recommended for lifeguards’ recovery after a water rescue.

## 1. Introduction

In recent years, several recovery tools with demonstrated benefits after exhausting efforts have transcended their common use in sports practice and rehabilitation [1,2] to other populations who also require a high level of physical conditioning (i.e., police or firefighters) [3,4]. These recovery methods aim to accelerate recovery and improve performance after an effort through passive and active techniques, including new devices with great potential [5,6]. In this vein, professional lifeguard teams have added these tools to their recovery routines after a water rescue, which is considered as a strenuous effort [5,6,7]. Prior investigations have pointed out the importance of recovery in lifeguards after a water rescue, since the safety of the rescuer and the survival of the victim are dependent on the lifeguard’s performance [8,9,10,11]. Considering that the drowning process usually occurs from 50 to 100 m from the shore, and with different conditions of waves or wind, lifeguards need to be concentrated and have a high level of physical conditioning [11,12], since the difficulty of the rescue can rapidly increase [13]. During a water rescue, lifeguards usually need to swim to the victim, control them and then tow them back to the shore [11,14]. Contrary to a sports practice or competition, when athletes know how much time they have to recover, on the beach lifeguards keep working after a water rescue, and the possibility of other events needs to be considered. Thus, it is of great importance for them to be physically prepared but also to recover quickly and properly [15].

One of the main consequences of a water rescue, in terms of physiological response, is the high level of blood lactate after the effort. Therefore, previous studies have analyzed the lifeguards’ recovery with traditional and novel techniques such as foam rollers or electrotherapy, with positive results [5,6,7]. These techniques have been demonstrated to enhance recovery by decreasing the blood lactate and the perceived fatigue after a water rescue [6,7]. Specifically, foam rollers and vibration foam rollers appear to be the most effective tools [6,7], with greater effects probably generated by the combination of pressure, movement and vibration over the tissues [16]. In recent years, a new method has been developed combining pressure, vibration and movement: percussive massage therapy (PMT). This method is applied by a therapeutic gun, which can be regulated in terms of vibration and provides an easy application over the body [17]. The combination of pressure, vibration and movement over the tissues has been demonstrated to influence the autonomic nervous system, increasing blood flow and modulating the muscle tone [16,18,19]. PMT has recently increased in popularity. Theoretically it could be an alternative for recovery in lifeguards after a water rescue, with a small economic investment and the possibility of applying it during vigilance. However, to date there have been few studies and little scientific evidence about PMT, and its effects on recovery still remain unclear [20,21]. Therefore, the aim of this study was to analyze the effects of PMT on lifeguards’ recovery after a water rescue, and to compare their effects with a passive recovery protocol.

## 2. Materials and Methods

**Figure 1:**
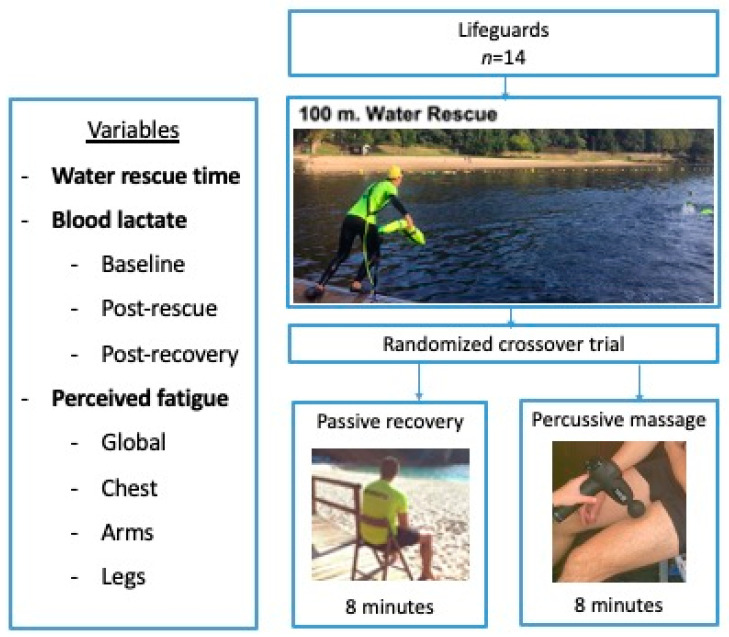
Flow chart outlining the design, intervention and variables.

A quasi-experimental crossover design was carried out in order to analyze the effects of PMT on lifeguards’ recovery after a water rescue, in comparison with a passive recovery. Specifically, the recovery variables selected were perceived fatigue and blood lactate clearance, according to previous studies [6,7]. The design and the intervention are detailed in Figure 1.

### 2.1. Sample

In this study, 14 lifeguards were recruited (90% men; age: 21.7 ± 2.0 years, weight: 72.9 ± 11.7 kg, height: 175.2 ± 9.5 cm; body mass index: 23.6 ± 2.1 kg/m^2^). The inclusion criteria were qualified lifeguards older than 18 years old and the exclusion criteria were injuries, chronic conditions or breathing diseases which could influence the rescue or the recovery. All of them were informed about the procedures and intervention of the study and voluntarily accepted to participate. All participants signed a written informed consent. This investigation was approved by the Ethics Committee of the Faculty of Education and Sport Sciences (03-1421) and developed according to the Declaration of Helsinki.

### 2.2. Procedures

In order to analyze the differences between passive recovery and PMT, two water rescues were performed by lifeguards on two different days, at the same time of the day and under similar conditions with calm sea, waves < 0.5 m (Douglas scale value 0–2), wind speed < 5 m/s, water temperature between 14 °C and 15 °C and ambient temperature between 17.5 °C and 19 °C in the river beach of Pontevedra, Galicia, Spain (latitude: 42.4222432, longitude: 8.6821066). The weather was reported by the local forecast agency (Meteogalicia). The water rescue was conducted with a test-approved water rescue manikin [22] with fins, wetsuits and rescue tube, and consisted of swimming 100 m towards the manikin, controlling the manikin, towing it 100 m back to the shore and extracting it to the dry sand [5,6,7,14]. At the end of the water rescue, lifeguards were asked to perform one of the recovery modalities selected, which were randomized.

### 2.3. Recovery

In this study, a comparison between two recovery modalities was performed: passive recovery vs PMT. Specifically, the passive recovery consisted of asking the participants to remain sitting after the water rescue for 8 min, simulating an immediate return to the watchtower. On the other hand, the PMT intervention consisted of applying a percussive massage with a gun (Backpack Pro, Backpack Physiosport, Madrid, Spain). This application was conducted by a physical therapist with knowledge and expertise in this method, and the muscles selected were quadriceps and hamstrings of both legs, since they have been reported to be highly involved in swimming with fins [23]. The intervention with PMT was performed for two minutes in each muscle, with a total time of 8 min. Specifically, the head of the gun was located at the end of the muscle and then it was compressed while moving the gun across the muscle belly. The frequency selected was 53 Hz, according to previous studies and the reference values for the musculoskeletal system [24]. In order to standardize the amount of pressure performed with the gun, a value of 6/10 in the numerical rating scale was used [25].

### 2.4. Variables

The time of the water rescue was recorded both days, in seconds. In order to analyze the perceived fatigue after the water rescue and after recovery, the Rating Perceived Exertion scale (RPE) was used. This scale uses values from 0 (no fatigue) to 10 (maximal fatigue) to rate the fatigue in 4 different areas: whole body, arms, chest and legs [6,7]. To analyze the physiological response to the effort and the consequent effects of recovery, blood lactate was measured in the finger. Each day, blood lactate was measured in three different moments: in baseline conditions (LB), after the water rescue (L1) and after recovery (L2). All measurements were performed with a capillary device (LactateScout, SensLab GmbH, Leipzig, Germany) and expressed in mmol/L, with an accuracy of ±3% (minimal standard deviation: ±0.2 mmol/L). Subjects were asked not to perform any exercise or training 24 h before the day of the test in order to avoid variations related to exercise in the measurements.

### 2.5. Statistical Analysis

All analyses were conducted using the statistical package SPSS for Macintosh (version 25.0, IBM Corp, Armonk, NY, USA). The normality of the distribution for each variable was checked both graphically and using the Shapiro–Wilk test. The descriptive results of these variables are reported as mean ± standard deviation (SD). A paired samples *t*-test was used to analyze the rescue time of participants according to each type of recovery. The effects of each recovery method on blood lactate and RPE variables were analyzed using repeated-measures analysis of variance (ANOVA) with two intra-subject factors (Recovery × Moment). Partial eta-squared (ηp^2^) effect sizes were also calculated for this analysis. A value ηp^2^ ≥ 0.01 indicates a small effect, ≥0.059 a medium effect and ≥0.138 a large effect [26]. Pair-wise comparisons were conducted via Bonferroni-adjusted post hoc test. For all analyses, the significance value was set at *p* ≤ 0.05.

## 3. Results

In this investigation, 14 lifeguards participated voluntarily and completed a total of 28 water rescues and then performed two different recovery modalities in a randomized order: passive recovery and PMT. The interventions were conducted on two different days, under similar conditions and with no significant differences in the rescue time between the two recovery modalities (passive recovery: 198.6 ± 24.1 s; PMT: 189.5 ± 28.0 s; t = 1.381, *p* = 0.190).

Regarding the perceived fatigue, analyzed with the RPE scale, results of the comparison of this variable between the two recovery methods are detailed in Table 1.

As shown in Table 1, global fatigue decreased significantly with passive recovery (*p* < 0.001) and with PMT (*p* < 0.001). The perceived fatigue of arms decreased significantly with passive recovery (*p* < 0.001) and with PMT (*p* < 0.001). In the chest, the perceived fatigue decreased significantly with passive recovery (*p* < 0.001) and with PMT (*p* < 0.001). Finally, in the legs, the perceived fatigue decreased significantly with passive recovery (*p* < 0.001) and with PMT (*p* < 0.001).

Results of blood lactate measurements are detailed in Table 2, comparing the three different moments with the two recovery modalities.

Results of the repeated-measures ANOVA showed significant differences between moments (*p* < 0.001; *n_p_*^2^ = 0.852, large) but not according to the type of recovery (*p* = 0.703; *n_p_*^2^ = 0.012, small) nor the interaction between moment and recovery (*p* = 0.922; *n_p_*^2^ = 0.006, trivial). In the pair-wise comparisons, passive recovery showed a significant increase of blood lactate in L2 (*p* < 0.001) and in L3 (*p* < 0.001) in comparison with L1. In PMT recovery, similar results were found with an increase of blood lactate in L2 (*p* < 0.001) and in L3 (*p* < 0.001) in comparison with L1.

## 4. Discussion

This study aimed to analyze the effects of PMT as a recovery tool in comparison with passive recovery in lifeguards after a 100 m water rescue. Findings in this investigation suggest that PMT does not enhance recovery after a water rescue in comparison with passive recovery, in terms of blood lactate and perceived fatigue.

PMT is as a novel technique with supposed benefits in recovery, specifically in the address of pain, perceived fatigue and the modulation of muscle tone. The benefits of PMT are suggested to be due to the combination of pressure, vibration and movement over the tissues, similar to other devices such as vibration foam rollers or roller massagers [16,19,25]. However, there is little evidence regarding PMT in comparison with foam rollers, and despite the fact that their benefits are supposed to be similar and generated by the same physiological mechanisms, the responses obtained on the tissues are clearly different. In this sense, there are no previous studies analyzing the effects of PMT on perceived fatigue, but with other methods such as foam rollers, vibration foam rollers and roller massagers this variable has been demonstrated to decrease significantly in sports population, healthy subjects and lifeguards after a water rescue [1,27,28]. Moreover, the foam roller with and without vibration has been shown to be an effective tool decreasing blood lactate, specifically after a simulated water rescue in lifeguards [6,7]. Therefore, although the foam rollers and the PMT appear to have similar mechanisms, in this study results suggest that the benefits may be different. In this regard, recovery with PMT was similar to the passive recovery. Considering that no previous studies analyzed the effects of PMT on perceived fatigue, it is difficult to compare them with similar interventions. Nevertheless, vibration foam rollers have been described to use similar mechanisms, with pressure added to vibration and movement over the tissues [29]. Contrary to our findings, vibration foam rollers and conventional foam rollers have reported a decrease on the perceived fatigue after the sports practice but also after a similar water rescue in lifeguards [6,7].

In the same vein, in this investigation the blood lactate clearance was similar in passive recovery and with PMT. Paying attention to the percentages of clearance, PMT appears to decrease slightly more blood lactate than passive recovery (9.6% vs. 8.1%). However, no statistical differences were found in this variable. Again, no prior research was developed analyzing the effects of PMT on blood lactate clearance. Nevertheless, with similar devices such as vibration foam rollers, blood lactate has been demonstrated to decrease significantly in comparison with passive recovery or even with conventional foam roller [7,30]. One possible explanation for these different findings could be the pressure exerted over the devices, since with foam rollers the bodyweight of the subjects is directly applied over the tool [16,31,32], but with the PMT the pressure is exerted by the physical therapist who perform the intervention [21]. In this sense, little has been studied about the effects of pressure in the tissue response of these type of interventions, but one of the main hypotheses suggests that higher pressures will contribute to achieve higher and faster effects [16,18,28]. This hypothesis is supported by the idea that the mechanoreceptors are involved in the first response of this recovery methods, and greater stimuli cause greater responses [33,34]. However, this hypothesis needs to be supported with further evidence.

On the other hand, different frequencies of vibration could also provide different results. The PMT can vibrate at different frequencies, and the frequency selected in this study was 53 Hz, according to previous investigations and within the range of influence of the musculoskeletal system (15–50 Hz) [35,36]. However, the results obtained with other devices such as vibration foam rollers were achieved with frequencies from 18 Hz to 25 Hz, which are quite far from the frequency used in the PMT. Prior researchers have hypothesized that higher frequencies added to the pressure and movement could be too much stimulation for the mechanoreceptors, especially the Pacini corpuscle and Golgi tendon organs, which would respond with no changes in the tissues involved [16,24,32]. In this sense, the participants of the study reported no harmful effects after the PMT recovery, but a prior study concluded that people using PMT should be cautious in this respect.

In this investigation, some limitations should be considered. First, considering that to date few studies have analyzed the effects of PMT, the lack of protocols and studies to compare with make results that should be read carefully. Second, the sample size is limited and the water rescue was simulated, but in a real situation results could differ from our findings. Despite the potential of this technique, this study does not show any advantages of using PMT in the recovery of lifeguards after a water rescue, in comparison with passive recovery or with other methods such as foam rollers. Future research should include PMT in recovery protocols, measuring different variables such as pain, perceived fatigue, blood lactate or other blood parameters in order to provide consistent results about the effects of this tool.

## 5. Conclusions

In conclusion, PMT does not seem to enhance recovery in lifeguards after a water rescue, in comparison with a passive recovery. Although PMT is a novel method with a high level of technology supporting it, results of this study do not support its use in lifeguard teams as a recovery tool to enhance blood lactate clearance or decrease perceived fatigue.

## Figures and Tables

**Table 1:** Results of the RPE scale (mean ± SD).

	Passive Recovery		PMT		ANOVA *p*-Value (*n_p_*^2^)		
	Post Rescue	Post Recovery	Post Rescue	Post Recovery	Recovery	Moment	Recovery × Moment
Global	7.5 ± 0.9	4.0 ± 1.6 *	7.0 ± 1.2	3.3 ± 1.8 *	0.080 (0.217)	<0.001 (0.917)	0.583 (0.024)
Arms	3.7 ± 1.6	1.4 ± 0.9 *	3.5 ± 1.8	1.4 ± 1.2 *	0.583 (0.024)	<0.001 (0.756)	0.551 (0.028)
Chest	7.3 ± 1.8	2.5 ± 1.7 *	7.1 ± 1.2	2.4 ± 2.3 *	0.773 (0.007)	<0.001 (0.891)	1.000 (0.000)
Legs	6.9 ± 1.2	3.6 ± 1.7 *	6.6 ± 1.5	2.9 ± 2.1 *	0.230 (0.109)	<0.001 (0.900)	0.444 (0.046)

PMT: percussive massage therapy; * Significant differences (*p* < 0.001) with post rescue.

**Table 2:** Blood lactate results in the three different moments (mean ± SD).

	L1	L2	L3
Passive recovery	3.6 ± 1.4	10.5 ± 2.7 *	9.7 ± 3.0 *
PMT	4.1 ± 1.7	10.8 ± 3.2 *	9.8 ± 3.1 *

PMT: percussive massage therapy; L1: pre rescue; L2: post rescue; L3: post recovery. * Significant difference (*p* < 0.001) with L1.

## Data Availability

Not applicable.
